# Alteration in topological properties of brain functional network after 2‐year high altitude exposure: A panel study

**DOI:** 10.1002/brb3.1656

**Published:** 2020-09-09

**Authors:** Zhenlong Xin, Xiaoming Chen, Qian Zhang, Jiye Wang, Yibin Xi, Jian Liu, Baojuan Li, Xiaoru Dong, Yiwen Lin, Wenbin Zhang, Jingyuan Chen, Wenjing Luo

**Affiliations:** ^1^ Department of Occupational and Environmental Health the Ministry of Education Key Lab of Hazard Assessment and Control in Special Operational Environment School of Public Health Air Force Medical University Xi'an China; ^2^ Department of Radiology Xijing Hospital Air Force Medical University Xi'an China; ^3^ Network Center Air Force Medical University Xi'an China; ^4^ School of Biomedical Engineering Air Force Medical University Xi'an China; ^5^ School of Basic Medical Science Peking University Beijing China

**Keywords:** brain functional network, cognition, high altitude, resting‐state functional MRI, topological properties

## Abstract

**Introduction:**

High altitude (HA) exposure leads to cognitive impairment while the underlying mechanism is still unclear. Brain functional network is crucial for advanced functions, and its alteration is implicated in cognitive decline in multiple diseases. The aim of current study was to investigate the topological changes in HA‐exposed brain functional network.

**Methods:**

Based on Shaanxi‐Tibet immigrant cohort, neuropsychological tests and resting‐state functional MRI were applied to evaluate the participants' cognitive function and functional connection (FC) changes, respectively. GRETNA toolbox was used to construct the brain functional network. The gray matter was parcellated into 116 anatomically defined regions according to Automated Anatomical Labeling atlas. Subsequently, the mean time series for each of the 116 regions were extracted and computed for Pearson's correlation coefficients. The relation matrix was further processed and seen as brain functional network. Correlation between functional network changes and neuropsychological results was also examined.

**Results:**

The cognitive performance was impaired by HA exposure as indicated by neuropsychological test. HA exposure led to alterations of degree centrality and nodal efficiency in multiple brain regions. Moreover, two subnetworks were extracted in which the FCs significantly decreased after exposure. In addition, the alterations in FCs within above two subnetworks were significantly correlated with changes of memory and reaction time.

**Conclusions:**

Our results suggest that HA exposure modulates the topological property of functional network and FCs of some important regions, which may impair the attention, perception, memory, motion ignition, and modulation processes, finally decreasing cognitive performance in neuropsychological tests.

## Graphical abstract

High altitude (HA) exposure modulates the topological property of functional network and functional connections (FCs) of some important regions, which may impair the attention, perception, memory, motion ignition, and modulation processes, finally decreasing cognitive performance in neuropsychological tests.

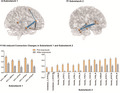

## 1 INTRODUCTION

High altitude (HA) exposure is becoming more and more common as the increased human activities in high plateaus and mountains. Most human, even those who lived in plateau for generations (Ebert‐Santos, 2017; Hill et al., 2016), are not totally adapted to HA exposure, as indicated by headache, sickness, poor sleep quality (Sakamoto et al., 2017), cognitive impairment (Chen et al., 2017), polycythemia, pulmonary hypertension (Robinson et al., 2017), right ventricular hypertrophy, and fertility reduction (Parraguez et al., 2013; Simonson, 2015; Whayne, 2014). With the huge demand of heath care for people exposed to HA, various studies were carried to explore mechanisms underlying HA‐induced health challenges. Notably, cognitive impairment was common especially in those undergoing long‐term HA exposure and had great influence on operation capability of people working under HA environment, while the pathophysiological mechanism underlying such HA‐induced alteration remains largely unclear.

Neuroimaging studies were widely employed in this field aiming at clarifying such phenomenon. Previous MRI studies have revealed some structural and functional changes after HA exposure which may partially explain the cognitive impairment. A previous study investigated 16 young man lived in high altitude for two years, revealing that HA subjects have significant changes of amplitude of low frequency fluctuation (ALFF), an indicator of spontaneous brain activity in the functional MRI signal, at bilateral occipital cortices, right anterior insula and extending to the caudate, putamen, inferior frontal orbital cortex, temporal pole, and superior temporal gyrus when compared with matched sea‐level natives (Zhang et al., 2017). Our previous study followed 69 participants who immigrated to Tibet for 2 years, finding that chronic HA exposure decreased regional homogeneity in the left putamen, superior temporal gyrus, superior parietal lobule, anterior cingulate gyrus, and medial frontal gyrus, while increased regional homogeneity was found in the hippocampus. Moreover, the functional connection (FC) between left putamen and superior temporal gyrus, anterior/middle cingulate gyrus, and other brain regions weakened after exposure (Chen et al., 2017).

Notably, human brain is particularly complex and different brain regions interact with each other to form a complicated network for advanced functions (Cao, Huang, & He, 2017). Among various network analysis approaches, graph theory analysis has been increasingly emphasized (Du et al., 2018). The graph theory analysis exacts mathematical representation of complex networks to form a framework comprised of nodes and edges. Each node represents a relatively independent brain region, and each edge refers to the FC among two nodes (Wang et al., 2015). The human brain network has some important topological properties which have been found to be associated with human neurological disorders, for example, degree centrality, a robust index of focal connectivity in which the number of direct connections from one node to all nodes is counted, reflecting its relative importance within a network (Yang, Dong, & Chawla, 2014). When compared with healthy controls, patients with bipolar disorder showed significant decrease in degree centrality in cortical regions including the middle temporal pole, inferior temporal gyrus, and ventral prefrontal cortex, which may be the underlying neural mechanisms in bipolar disorder (Zhou et al., 2017). The nodal efficiency of the network refers to the averaged reciprocal shortest path length between the node and the other nodes, representing the ability of information transfer from itself to other nodes in the entire network (Achard & Bullmore, 2007). Similarly, the alteration in nodal efficiency is also observed in multiple psychiatric and neurological disorders. Based on previous studies, it is intriguing to investigate whether HA exposure leads to topological abnormalities in the functional brain networks and whether such abnormalities are correlated with cognitive decline.

Our previous study found that some connections between different brain regions were changed after HA exposure by using seed‐based analysis (Chen et al., 2017). However, there was still no study about the changes in FCs from a perspective of whole brain networks. Notably, in current study, we constructed the functional brain networks and compared the topological properties before and after exposure. We hypothesized that chronic HA exposure may alter the topological properties in cognitive‐related brain regions and FCs among these regions would be weakened. Moreover, the topological and FC changes may be correlated with HA‐induced neuropsychological changes. To the best of our knowledge, this is the first study focusing on the chronic HA exposure induces brain functional network changes through graph‐based theoretical approaches.

## 2 METHODS

### 2.1 Participants

The study was ratified by the Ethics Committee of the Medical Faculty of Air Force Medical University (No. KY20143344‐1), and written informed consent was obtained from each participant. We always abided by the Declaration of Helsinki during the whole process of research.

All 69 participants in current study came from Shaanxi‐Tibet immigrant cohort (STI cohort). The detailed information about this cohort has been described in our previous article (Chen et al., 2017). The 69 young healthy high school graduates from Shaanxi (400 m) were admitted into Tibet University (3,650 m) for higher education and recruited to the STI cohort study which was launched in 2014.

Firstly, the information about HA exposure history, medical history, and sociodemographic status (parental education, vocation, socioeconomic status, etc.) was collected at baseline. Moreover, the participants received neuropsychological investigation and MRI measure before and after a 2‐year HA experience. Finally, there were 49 participants completed the neuropsychological test and MRI examination both in 2014 and 2016.

### 2.2 Neuropsychological test

We conducted four tests to monitor the memory ability and reaction of each participant, including (a) verbal memory test, which was design to measure working memory for words, including the examination for immediate and delayed verbal memory; (b) visual memory test, which was employed to measure working memory for figures and shapes, aiming at testing immediate visual memory and delayed visual memory; (c) simple reaction time test, which was employed to assess psychomotor function in response to a simple stimulus, testing visual simple reaction time and auditory simple reaction time; and (d) recognition (go/no‐go) reaction time test was applied to measure psychomotor function in response to target/ distractive stimuli, thereby testing visual recognition reaction time and auditory recognition reaction time. All tests were performed using CNS Vital Signs (http://www.cnsvs.com/). Moreover, above neuropsychological tests were performed just before MRI measurement to better correlate the neuropsychological test results with neuroimaging data. The original data of cognitive performance can be seen in online Supporting Information.

### 2.3 MRI data acquisition

Functional MRI data were acquired with General Electric Discovery MR750 3.0T (General Electric Co. Ltd.) in Xijing Hospital of the Fourth Military Medical University (2014) and the General Hospital of Tibet Military Region (2016), respectively.

We strictly employ the same MRI parameters in the whole process in order to ensure the comparability of the images. Standard T1‐weighted 3D anatomical data were acquired with the 3D magnetization‐prepared rapid gradient echo (3D MPRAGE) sequence (repetition time: 2,530 ms; echo time: 3.5 ms; flip angle: 7°; field of view: 256 mm × 256 mm; matrix: 256 × 256; slice thickness: 1 mm; section gap: 0 mm; number of slices: 192; and voxel size = 1 × 1 × 1 mm^3^).

The following echo‐planar imaging (EPI) sequence which covers the entire brain was applied during resting‐state functional MRI data acquirement: repetition time: 2,000 ms; echo time: 30 ms; flip angle: 90°; field of view: 220 mm × 220 mm; acquisition/reconstruction matrix: 128 × 128; slice thickness: 4 mm; section gap: 0.6 mm; number of slices: 30, voxel size = 1 × 1 × 1 mm^3^, scam time: 6 min; and total volumes: 180. We use earplugs and custom‐built head coil cushions to dampen scanner noise and minimize head motion. During data acquisition, subjects were asked to remain alert with eyes closed and to keep their head still.

### 2.4 Data preprocessing

The fMRI data were preprocessed using GRETNA (v2.0) (GRETNA, RRID:SCR_009487) (Wang et al., 2015). In summary, the preprocessing includes (a) converting DICOM files to NIfTI format; (b) discarding the first 10 volumes of NIfTI format; (c) slice‐timing correction; (d) the functional MRI data were realigned to correct for head motion; (e) the T1 images were further utilized for normalization and segmentation, and each subjects' brain image was segmented into gray matter, white matter, and cerebrospinal fluid; (f) functional images were normalized to standard MNI space and smoothed with a 6‐mm full width at half maximum Gaussian kernel; (g) linear detrend and band‐pass filtering (0.01–0.08 Hz) were conducted; and (h) regressing several nuisance signals including head motion, global mean, and signals from the cerebrospinal fluid and white matter from the data. For the before exposure group, six subjects were excluded for some reasons: one for the artifact of T1 images, four for excessive head motion, and another one for unsatisfied normalization. For the after exposure group, 10 subjects were also excluded for some reasons: one for the artifact of functional images, four for excessive head motion, and five for unsatisfied normalization.

### 2.5 Brain network construction

GRETNA, a graph theoretical network analysis toolbox for imaging connectomics, was employed for construction of functional brain network (Wang et al., 2015). The whole cerebral and cerebellar gray matter was parcellated into 116 anatomically defined regions according to Automated Anatomical Labeling (AAL) atlas. Subsequently, the mean time series for each of the 116 regions were extracted and computed for Pearson's correlation coefficients. Fisher's *z* transformation was performed to turn the data into a *z*‐value which was close to a normal distribution. Moreover, the graphic model of the brain functional network was constructed by a binary connection matrix, which was converted by the *z*‐values with a series of threshold (Sparsity: 5%–50%) of the relation matrix.

### 2.6 Global and nodal network properties

The brain network is seen as a graph in math. The mathematic study of graph is named as graph theory. In this study, applying graph theory to brain network, each brain region is defined as a node and each connection between two nodes is defined as an edge in graph. Thereby, the network property can be quantified by some indexes in graph theory. Generally, these indexes can be categorized from global and nodal perspectives.

Following indexes reflect global network property. Small‐world networks have a shorter characteristic path length and greater local interconnectivity, which maximize the efficiency of information transfer at a relatively low wiring cost. Global efficiency measures the global efficiency of parallel information transfer in a network. The local efficiency of the network measures how efficient communication is among the first neighbors of a given node when it is removed. Rich‐club architecture indicates that the hub nodes are more densely connected among themselves than nonhub nodes and thus form a highly interconnected club. Assortativity reflects the tendency of nodes to link those nodes with similar numbers of edges. Synchronization measures how likely it is that all nodes fluctuate in the same wave pattern. The hierarchy coefficient is used to identify the presence of a hierarchical organization in a network.

Some indexes reflect the role of a given nodal in the network. Degree of a given node means the number of edges connecting it to the rest of the graph. It is easy to understand that there are many paths between two nodes in graph. The optimal path which has minimal edge numbers is named as shortest path length. For two brain regions, it is easier to communicate with each other when the shortest path length is smaller. The efficiency of a given node is inversely proportional to its shortest path length to other nodes in whole graph. Besides, the local efficiency for a given node measures how efficient the communication is among the first neighbors of this node when it is removed. The centrality captures the importance of a given node (Hagmann et al., 2008), which can be calculated from degree or betweenness. A node with higher degree also has higher degree centrality, which reflects its information communication ability in graph. Moreover, a node with high betweenness centrality lies on many of the shortest paths that link other nodes in the network to one another, so it has significant impact on information flow between other nodes. The clustering coefficient of a given node measures the likelihood its neighborhoods are connected to each other. Modularity refers to the existence of multiple densely connected communities of regions in a brain network. The participant coefficient reflects the ability of an index node in keeping communication between its own module and the other modules.

For more details about formula, usage, and interpretation of these indexes, please refer to Rubinov and Sporns (2010), Wang et al. (2011), and Reference Manual for GRETNA (v2.0).

### 2.7 Statistical analysis

To determine the significant before–after differences of all the network matrices (global and nodal properties and the AUC of each network metric), paired *t* test analysis was used in current study. FDR correction of *p* < .05 was applied to control for multiple comparisons in nodal properties. The internodal connections were also compared by paired *t* test. Network‐based statistic (NBS) was used to control the error rate (Edge *p* = .0001, Component *p* = .01, Number of iterations = 1,000). Moreover, Pearson's correlation was performed to determine the relationship between brain functional network alterations and neuropsychological changes. The statistical analyses were performed with GRETNA and GraphPad (GraphPad Prism, RRID:SCR_002798). *p* < .05 was considered statistically significant.

## 3 RESULTS

### 3.1 HA exposure induced cognitive function impairment

The current study enrolled young adults who immigrated to HA region for higher education. We followed them for 2 years aiming at evaluating HA‐induced cognitive and brain functional network alteration. The result of neuropsychological tests revealed that HA exposure decreased accuracy in the verbal/visual memory test and prolonged responding time in the visual/auditory reaction time test. The details about cognitive function impairment were described elsewhere (Chen et al., 2017).

### 3.2 All global network properties remained intact after exposure

Global metrics we focused in this study included small‐world parameters, local efficiency and global efficiency, modularity, rich club, assortativity, synchronization, and hierarchy. By using GRETNA, we calculated and compared these metrics before and after exposure. As a result, there were no significant changes among all global network properties.

### 3.3 Degree centrality and nodal efficiency changed in some regions after exposure

**FIGURE 1:**
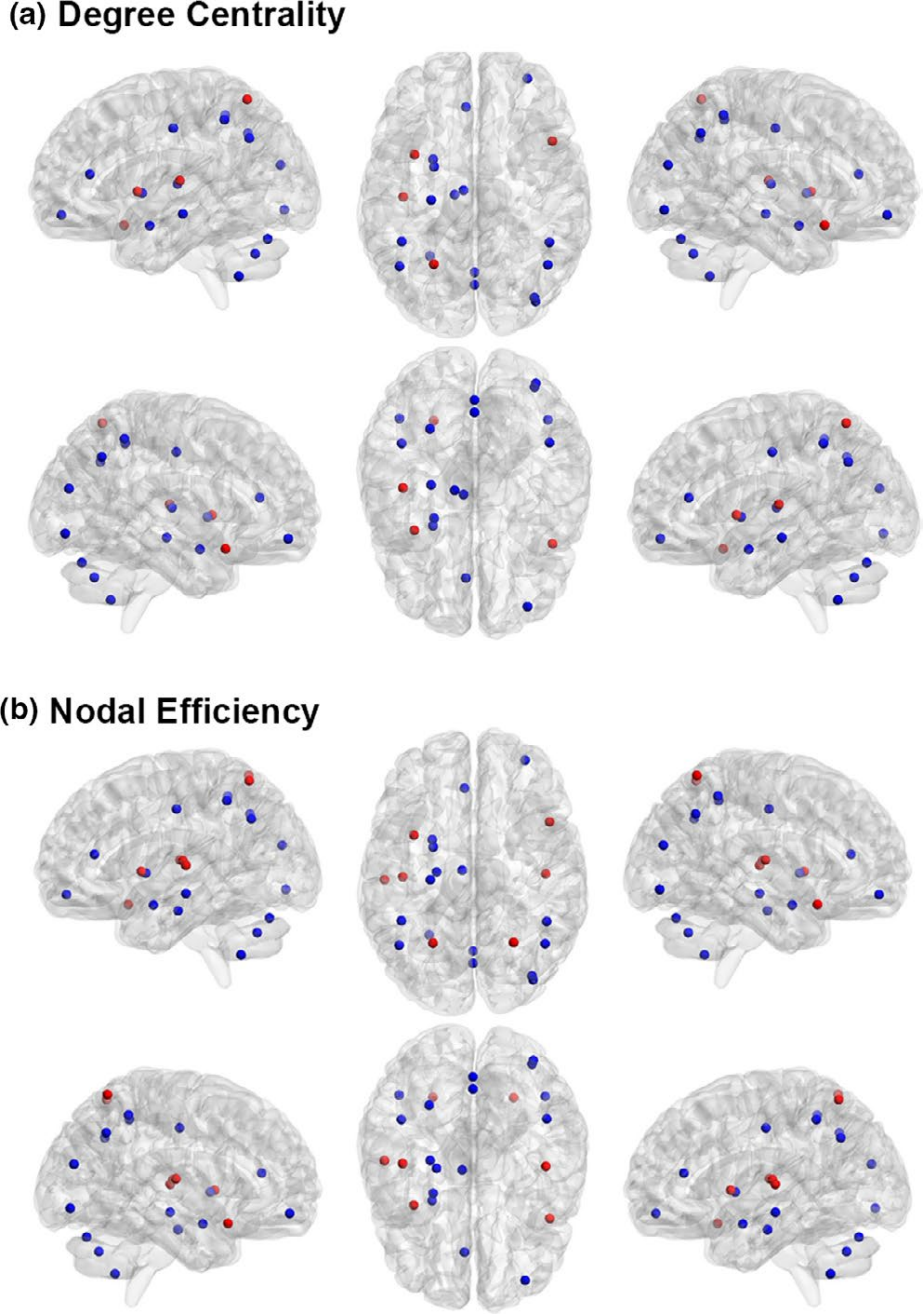
The changes of regional nodal properties after high altitude (HA) exposure. Nodes with increased degree centrality or nodal efficiency are shown in red. Nodes with decreased degree centrality or nodal efficiency are shown in blue. The figure is made by Brain Net Viewer (http://www.nitrc.org/projects/bnv/). (a) Degree centrality and (b) node efficiency

Alterations in degree centrality were revealed in 20 brain regions (|*t*| > 2.9878, *p* < .05 FDR corrected). Among them, the degree centrality of 12 regions decreased and eight regions elevated after exposure. See details in Figure 1 and Table 1.

**TABLE 1:** Degree centrality changes after HA exposure

Nodes	*T* value (*T* threshold = 2.9878)	Trends
Frontal_Mid_Orb_R	−3.2098	**↑**
Insula_L	3.4133	**↓**
Cingulum_Ant_L	3.5814	**↓**
Cingulum_Mid_L	3.4721	**↓**
Hippocampus_L	4.0025	**↓**
Amygdala_L	3.2161	**↓**
Occipital_Mid_R	−3.5138	**↑**
Occipital_Inf_R	−4.0754	**↑**
Parietal_Sup_L	−3.2968	**↑**
Parietal_Inf_L	−4.2666	**↑**
Parietal_Inf_R	−3.2948	**↑**
Angular_L	−3.5115	**↑**
Angular_R	−3.6746	**↑**
Putamen_L	3.1082	**↓**
Thalamus_L	3.0585	**↓**
Heschl_L	3.6709	**↓**
Temporal_Pole_Sup_R	3.0798	**↓**
Cerebelum_8_L	3.0178	**↓**
Vermis_7	4.0659	**↓**
Vermis_8	3.5644	↓

Abbreviations: Amygdala_L, left amygdala; Angular_L, left angular; Angular_R, right angular; Cerebelum_8_L, left cerebelum_8; Cingulum_Ant_L, left anterior cingulate gyrus; Cingulum_Mid_L, median anterior cingulate gyrus; Frontal_Mid_Orb_R, right middle frontal gyrus, orbital; Heschl_L, left Heschl; Hippocampus_L, left hippocampus; Insula_L, left insula; Occipital_Inf_R, right inferior occipital gyrus; Occipital_Mid_R, right middle occipital gyrus; Parietal_Inf_L, left inferior parietal gyrus; Parietal_Inf_R, right inferior parietal gyrus; Parietal_Sup_L, left superior parietal gyrus; Putamen_L, left putamen; Temporal_Pole_Sup_R, superior temporal gyrus temporal pole; Thalamus_L, left thalamus.

Alterations in nodal efficiency were revealed in 23 brain regions (|*t*| > 2.8255, *p* < .05 FDR corrected). Among them, the nodal efficiency of 14 regions decreased and nine regions elevated after HA exposure. See details in Figure 1 and Table 2.

**TABLE 2:** Nodal efficiency changes after HA exposure

Nodes	*T* value (*T* threshold = 2.8255)	Trends
Frontal_Mid_Orb_R	−3.0778	**↑**
Insula_L	3.6251	**↓**
Cingulum_Ant_L	3.3516	**↓**
Cingulum_Mid_L	3.3371	**↓**
Hippocampus_L	4.3508	**↓**
ParaHippocampal_L	3.0820	**↓**
Amygdala_L	3.5881	**↓**
Occipital_Mid_R	−3.5108	**↑**
Occipital_Inf_R	−3.6796	**↑**
Parietal_Sup_L	−3.1712	**↑**
Parietal_Sup_R	−3.2178	**↑**
Parietal_Inf_L	−4.1644	**↑**
Parietal_Inf_R	−2.8886	**↑**
Angular_L	−3.3717	**↑**
Angular_R	−3.4027	**↑**
Putamen_L	2.8672	**↓**
Heschl_L	3.9777	**↓**
Heschl_R	3.0264	**↓**
Temporal_Sup_L	3.1168	**↓**
Temporal_Pole_Sup_R	3.5580	**↓**
Cerebelum_8_L	3.2812	**↓**
Vermis_7	3.9301	**↓**
Vermis_8	3.2520	**↓**

Abbreviations: Amygdala_L, left amygdala; Angular_L, left angular; Angular_R, right angular; Cerebelum_8_L, left cerebelum_8; Cingulum_Ant_L, left anterior cingulate gyrus; Cingulum_Mid_L, median anterior cingulate gyrus; Frontal_Mid_Orb_R, right middle frontal gyrus, orbital; Heschl_L, left Heschl; Heschl_R, right Heschl; Hippocampus_L, left hippocampus; Insula_L, left insula; Occipital_Inf_R, right inferior occipital gyrus; Occipital_Mid_R, right middle occipital gyrus; ParaHippocampal_L, left parahippocampal gyrus; Parietal_Inf_L, left inferior parietal gyrus; Parietal_Inf_R, right inferior parietal gyrus; Parietal_Sup_L, left superior parietal gyrus; Putamen_L, left putamen; Temporal_Pole_Sup_R, superior temporal gyrus temporal pole; Temporal_Sup_L, left superior temporal gyrus.

### 3.4 FCs within two subnetworks changed after exposure

**FIGURE 2:**
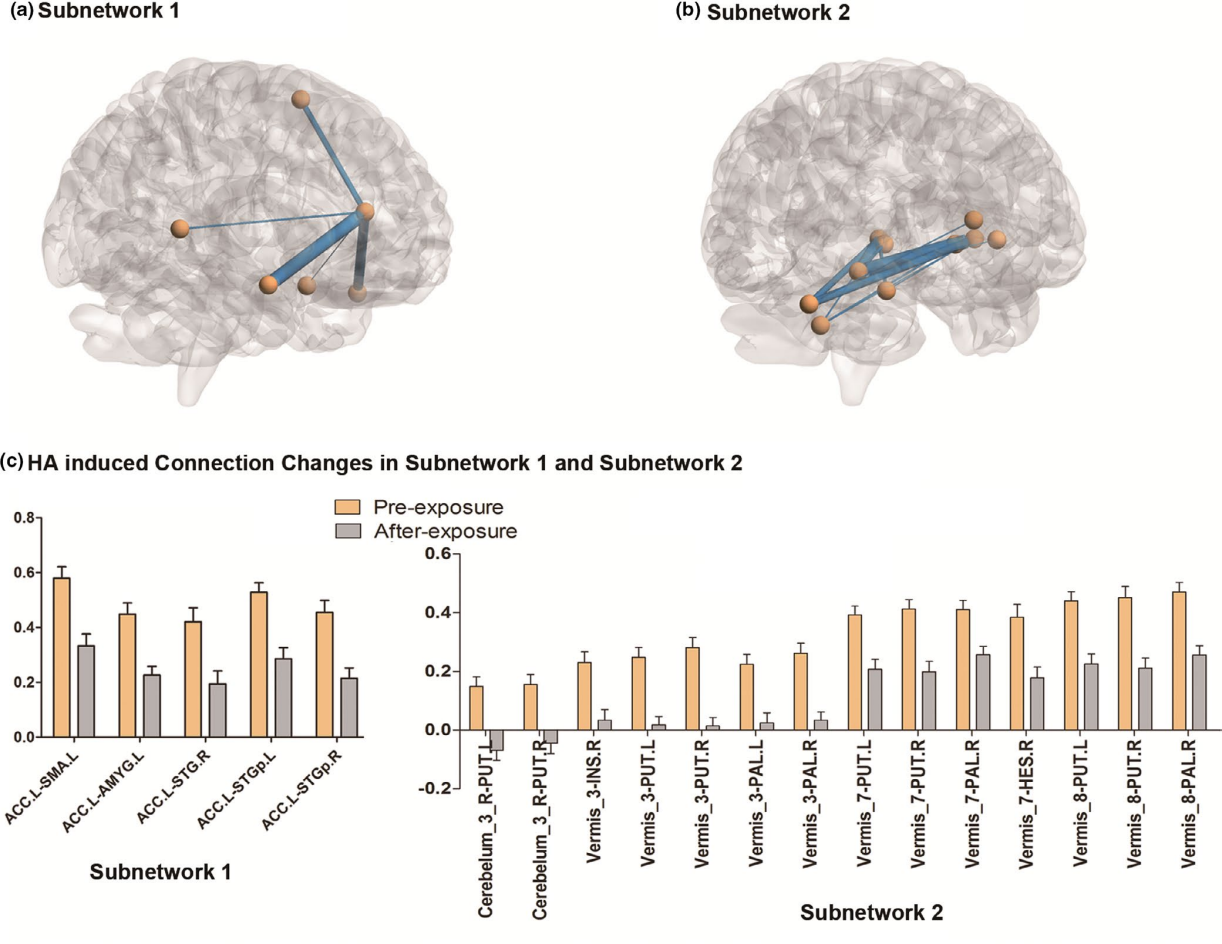
The changed subnetworks after high altitude (HA) exposure. (a) Subnetwork 1 extracted from whole brain functional network. (b) Subnetwork 2 extracted from whole brain functional network. (c) The functional connections in each subnetwork significantly decreased after exposure. ACC.L, left anterior cingulate gyrus; AMYG.L, left amygdala; HES.R, right Heschl's gyrus; INS.R, right insula; PAL.L, left pallidum; PAL.R, right pallidum; PUT.L, left putamen; PUT.R, right putamen; SMA.L, left supplementary motor area; STG.R, right superior temporal gyrus; STGp.L, left superior temporal gyrus temporal pole; STGp.R, right superior temporal gyrus temporal pole

Apart from above network metrics, some FCs were also changed in response to HA challenges. NBS correction revealed two subnetworks with statistically significant changes (Figure 2 and Table 3). Notably, all FCs were decreased in both subnetworks (Figure 2).

**TABLE 3:** Subnetworks with changed functional connections (FCs) after HA exposure

Subnetwork	Brain regions with FC	Trends
Subnetwork 1		
Cingulum_Ant_L	Supp_Motor_Area_L	**↓**
Cingulum_Ant_L	Amygdala_L	**↓**
Cingulum_Ant_L	Temporal_Sup_R	**↓**
Cingulum_Ant_L	Temporal_Pole_Sup_L	**↓**
Cingulum_Ant_L	Temporal_Pole_Sup_R	**↓**
Subnetwork 2		
Cerebelum_3_R	Putamen_L	**↓**
Cerebelum_3_R	Putamen_R	**↓**
Vermis_3	Insula_R	**↓**
Vermis_3	Putamen_L	**↓**
Vermis_3	Putamen_R	**↓**
Vermis_3	Pallidum_L	**↓**
Vermis_3	Pallidum_R	**↓**
Vermis_7	Putamen_L	**↓**
Vermis_7	Putamen_R	**↓**
Vermis_7	Pallidum_R	**↓**
Vermis_7	Heschl_R	**↓**
Vermis_8	Putamen_L	**↓**
Vermis_8	Putamen_R	**↓**
Vermis_8	Pallidum_R	**↓**

Abbreviations: ACC.L, left anterior cingulate gyrus; AMYG.L, left amygdala; HES.R, right Heschl's gyrus; INS.R, right insula; PAL.L, left pallidum; PAL.R, right pallidum; PUT.L, left putamen; PUT.R, right putamen; SMA.L, left supplementary motor area; STG.R, right superior temporal gyrus; STGp.L, left superior temporal gyrus temporal pole; STGp.R, right superior temporal gyrus temporal pole.

### 3.5 Significant correlations between brain network alterations and cognitive changes

**FIGURE 3:**
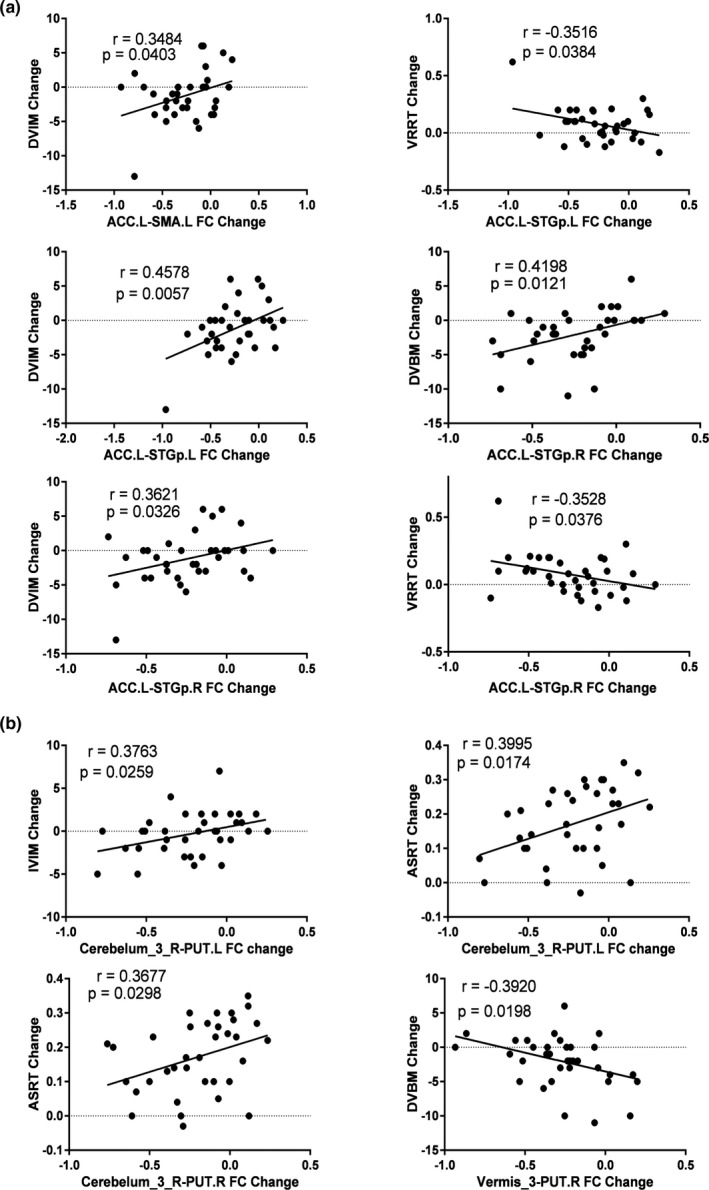
The correlation between functional connection (FC) decrease and cognitive impairment. Scatter plots indicating the correlation between the HA‐induced FC changes and corresponding cognitive performance changes (*p* < .05) in subnetwork 1 (a) and subnetwork 2 (b). ASRT, auditory simple reaction time; DVBM, delayed verbal memory; DVIM, delayed visual memory; IVIM, immediate visual memory; PUT.L, left putamen; PUT.R, right putamen; SMA.L, left supplementary motor area; STGp.L, left superior temporal gyrus temporal pole; STGp.R, right superior temporal gyrus temporal pole; VRRT, visual recognition reaction time

To understand the effects of HA exposure on brain network and function, we performed correlation analyses between brain network alterations and cognitive decline. Notably, some alterations in FCs within above two subnetworks were associated with changes of memory and reaction time (Figure 3a,b, especially). In subnetwork 1, the decline of delayed visual memory (DVIM) was positively associated with decrease in FCs between left anterior cingulate gyrus and left supplementary motor area (*p* = .0403, *r* = .3484), left superior temporal gyrus temporal pole (*p* = .0057, *r* = .4578), and right superior temporal gyrus temporal pole (*p* = .0326, *r* = .3621). The accuracy change in delayed verbal memory (DVBM) was positively associated with decrease in FC between left anterior cingulate gyrus and right superior temporal gyrus temporal pole (*p* = .0121, *r* = .4198). The prolonged visual recognition reaction time (VRRT) was negatively associated with changed FCs between left anterior cingulate gyrus and left superior temporal gyrus temporal pole (*p* = .0384, *r* = .3516), and right superior temporal gyrus temporal pole (*p* = .0376, *r* = .3528). In subnetwork 2, the FC decrease between right cerebelum_3 and left putamen was positively correlated with alteration in immediate visual memory (IVIM; *p* = .0259, *r* = .3763) and auditory simple reaction time (ASRT) (*p* = .0174, *r* = .3995). The prolonged ASRT was also positively associated with FC change between cerebelum_3 and right putamen (*p* = .0298, *r* = .3677). In addition, the DVBM decline was negatively associated with attenuated FC between vermis_3 and right putamen (*p* = .0198, *r* = .3920).

## 4 DISCUSSION

High altitude‐induced cognitive impairment is an important public health problem remaining to be solved in HA regions. Extensive efforts have been made to clarify the underlying mechanisms while limited progress has been made in past years. In this study, by investigating HA‐induced changes in topological properties of brain functional networks, we revealed some topological and FC changes after two‐year HA challenge, which may be the mechanism underlying HA‐induced cognitive impairment.

First of all, there were no significant changes in global metrics which include small‐world parameters, local efficiency, global efficiency, modularity, rich club, assortativity, synchronization, and hierarchy. Such phenomenon may suggest that two‐year HA exposure was not enough to cause topological properties changes from global perspectives.

However, according to our results, some nodal metrics were significantly altered after two‐year HA exposure (Tables 1 and 2). Twenty brain regions were observed to have changes with regard to degree centrality, and 23 brain regions changed in nodal efficiency. Notably, nearly all brain regions with changed degree centrality also have nodal efficiency alteration. Moreover, the alteration in nodal degree and efficiency was consistent in each region.

Some brain regions were found to have both decreased degree centrality and nodal efficiency, including left insula, left anterior cingulate gyrus, left median cingulate gyrus, left hippocampus, left amygdala, left putamen, left Heschl, right temporal pole, left cerebelum_8, vermis_7, and vermis_8. The left insula, left anterior cingulate gyrus and left median cingulate gyrus all belong to default mode network which is tightly associated with memory and reaction (Barber, Caffo, Pekar, & Mostofsky, 2017; Gotting et al., 2017; Nelson et al., 2016; Staffaroni et al., 2018). Moreover, the left anterior cingulate gyrus plays an important role in attentional control. The altered FCs between left anterior cingulate gyrus and multiple brain regions were associated with decreased attention performance in the multiple sclerosis patients (Loitfelder et al., 2012). The left hippocampus and left amygdala are typical regions implicated in managing memories. Activity change or damages in these regions will certainly influence memory performance (Claire, Sophie, Claudia, Philippe, & Eliane, 2016; Krause‐Utz et al., 2018; Witt et al., 2014). The right temporal pole was reported to not only influence spatial and nonspatial short‐term memory but also involved in sound localization discrimination or auditory object discrimination (Lancelot et al., 2003). The cerebellum, which is crucial for motor coordination, is also influenced after HA exposure. Collectively, two‐year HA exposure decreased the degree centrality and nodal efficiency of some crucial regions that regulate attention control, reaction, memory, and motor coordination, finally leading to worse performance in neuropsychological tests. Notably, most of abovementioned regions located in the left hemisphere, suggesting that left hemisphere is more vulnerable under hypoxic condition, which is consistent with a previous study (Yoo, Shin, Chang, & Caplan, 1998). The difference of vascular structure between left and right hemisphere may account for such left‐preferring phenomenon. In pre‐ and postmortem studies, the mean vertebral artery diameter was found to be larger on the left side than the right side (Khan, Cloud, Kerry, & Markus, 2007). So we speculate that left hemisphere may be accustomed to massive blood and oxygen supply, which may make it more vulnerable to hypoxic condition.

The degree centrality and nodal efficiency increased in several brain regions, including right orbital middle frontal gyrus, right middle occipital gyrus, right inferior occipital gyrus, left superior parietal gyrus, bilateral inferior parietal gyri, and bilateral angular gyri. Most of these regions belong to association cortex which is an evolutionarily recent type of cortex for advanced information processing related to cognition (Khodagholy, Gelinas, & Buzsaki, 2017). Association regions integrate multiple functional systems, such as memory and attention systems, and are mainly involved in intelligent processing (Mesulam, 1998). We inferred that the improvement in association cortices could be a compensation to the decline of some nodal metrics in cognition‐related regions.

Apart from these, two changed subnetworks were extracted after exposure. In subnetwork 1, the left anterior cingulate gyrus served as a central node. The FCs between left anterior cingulate gyrus and five regions (including left supplementary motor area, left amygdala, right superior temporal gyrus, and bilateral temporal pole) significantly decreased after HA exposure. As a highly concerned brain region, the left anterior cingulate gyrus frequently served as a hub in multiple studies, which may help to integrate various signals from multiple sources. The bilateral anterior temporal lobes responded to environmental sound stimuli and played important roles in semantic processes (Visser & Lambon Ralph, 2011). Moreover, as a recent study revealed, the left anterior temporal lobe and anterior cingulate cortex were semantic hub regions, whose damage was significantly correlated with the severity of the deficit in semantic performance (Zhao et al., 2017). Thereby, we propose that the decrease in FCs between left anterior cingulate gyrus and bilateral temporal lobes may impair the auditory perception and comprehension, finally influencing performance in neuropsychological tests. In addition, the FCs of motor and memory‐associated regions also decreased, which influence the test score from other aspects. In subnetwork 2, the hubs turned to be some cerebellar regions, including parts of cerebellar hemisphere and vermis. The other sides of these connections are components of bilateral basal ganglia. As is well known, the cerebellum and basal ganglia function as two pivotal regions for motor coordination. The lesions occurred in cerebellum, and basal ganglia could lead to motor coordination impairment, such as cerebellar ataxia (Slapik et al., 2019) and Parkinson's disease (Rolinski et al., 2016). Moreover, recent studies revealed that cerebellum and basal ganglia interacted with each other during predictive motor timing which is crucial flexible movement adjustment (Caligiore et al., 2017; Husárová et al., 2013; Kunimatsu, Suzuki, Ohmae, & Tanaka, 2018). Thereby, the subnetwork 2 mainly connects two motor coordination regions, namely cerebellum and basal ganglia, bearing the responsibility of motion response in neuropsychological tests. Thereby, the HA‐induced FCs decrease in subnetwork 2 may be associated with the slower response in simple reaction time task from a motor execution perspective. What's more, the alterations in FCs within above two subnetworks were significantly correlated with changes of memory and reaction time.

As shown in Part A of Figure 3, the memory score changes positively correlated with FC changes, so the decreases of FC in subnetwork 1 may account for the decreases in memory score. The changes of VRRT negatively correlated with FC changes, so the decreases of FC in subnetwork 1 may account for prolonged VRRT. Similarly, as shown in Part B of Figure 3, the IVIM change positively correlated with FC change between right cerebelum_3 and left putamen, so the decrease in this FC may account for the decrease in IVIM. However, other correlations in Part B of Figure 3 were relatively difficult to understand. The FC changes between right cerebelum_3 and bilateral putamen positively correlated with ASRT change, which indicated that HA induced these FC decreases may shorten ASRT. However, actually, the ASRT significantly prolonged after HA exposure. We speculate that the prolonged ASRT may be caused by other alteration, like decreased regional homogeneity in right superior temporal gyrus (Chen et al., 2017). The alterations in FC between right cerebelum_3 and bilateral putamen may be inadequately compensatory responses to HA‐induced ASRT prolongation. Moreover, the DVBM change negatively correlated with FC change between vermis_3 and right putamen. Similarly, this decreased FC may also be an inadequately compensatory response to HA‐induced memory decline. However, such speculations still need to be examined in future studies.

Notably, there are some limitations regarding current study. First of all, this is a self‐control study aiming at clarifying HA‐induced brain functional network changes, while it lacks a sea‐level control cohort to be more rigorous. We plan to establish a sea‐level college student cohort in future studies. Second, this study only included young adults while other people who also endure HA challenges in HA regions, like children, adults, and the olders were not considered in current study. Thereby, further studies may focus on different population and reveal the alterations in their brain functional networks after HA exposure. What's more, the AAL template was employed in current study to define the nodes of functional network. However, this template is not fine enough for its relative low resolution. The more comple x templates, like probabilistic atlas, should be applied in future studies.

## 5 CONCLUSION

In our current study, we constructed the functional brain network and compared the topology properties before and after 2‐year HA exposure. We found that HA‐exposed young adults exhibited altered degree centrality and nodal efficiency in multiple brain regions which were involved in attention control, reaction, memory, and motor coordination. Moreover, two changed subnetworks were extracted after exposure. The subnetwork 1 connected brain regions controlling auditory perception, comprehension, motion initiation, and memory. The subnetwork 2 connected the two motor coordination system, which helps to regulate motion response in neuropsychological tests. In a word, through modulating the topological properties of functional network and FCs of some important regions, HA exposure may impair the attention, perception, memory, motion ignition, and modulation processes, finally decreasing cognitive performance in neuropsychological tests.

## CONFLICT OF INTEREST

The authors declare no conflict of interest.

## AUTHORS' CONTRIBUTIONS

W‐JL, J‐YC, and W‐BZ contributed equally to the conception and design of the study. Z‐LX and X‐MC were responsible for data processing and manuscript drafting. Z‐LX, X‐MC, QZ, J‐YW, and Y‐BX collected MRI and cognition data. QZ, J‐YW, JL, B‐JL, and X‐RD contributed to the data analysis. All authors revised and approved the final draft of this article.

## ETHICAL APPROVAL

The study was ratified by the Ethics Committee of the Medical Faculty of Air Force Medical University (No. KY20143344‐1), and written informed consent was obtained from each participant. We always abided by the Declaration of Helsinki during the whole process of research.

## Supporting information

Supplementary Material

## Data Availability

The data that support the findings of this study are available from the corresponding author upon reasonable request.
